# Transcriptional regulation of plant innate immunity

**DOI:** 10.1042/EBC20210100

**Published:** 2022-09-30

**Authors:** Niels Aerts, Himanshu Chhillar, Pingtao Ding, Saskia C.M. Van Wees

**Affiliations:** 1 Plant-Microbe Interactions, Department of Biology, Science4Life, Utrecht University, 3508 TB Utrecht, The Netherlands; 2 Institute of Biology Leiden, Leiden University, 2333 BE Leiden, The Netherlands

**Keywords:** gene expression and regulation, plant immunity, RNA, transcription factors

## Abstract

Transcriptional reprogramming is an integral part of plant immunity. Tight regulation of the immune transcriptome is essential for a proper response of plants to different types of pathogens. Consequently, transcriptional regulators are proven targets of pathogens to enhance their virulence. The plant immune transcriptome is regulated by many different, interconnected mechanisms that can determine the rate at which genes are transcribed. These include intracellular calcium signaling, modulation of the redox state, post-translational modifications of transcriptional regulators, histone modifications, DNA methylation, modulation of RNA polymerases, alternative transcription inititation, the Mediator complex and regulation by non-coding RNAs. In addition, on their journey from transcription to translation, mRNAs are further modulated through mechanisms such as nuclear RNA retention, storage of mRNA in stress granules and P-bodies, and post-transcriptional gene silencing. In this review, we highlight the latest insights into these mechanisms. Furthermore, we discuss some emerging technologies that promise to greatly enhance our understanding of the regulation of the plant immune transcriptome in the future.

## Introduction

Plant diseases caused by different pathogens pose a major threat to crop productivity. However, plants do respond to pathogens by activating their robust yet specialized innate immune system. General pathogen-associated molecular patterns (PAMPs) and specific apoplastic pathogen effectors are perceived by the plants’ surface-localized pattern-recognition receptors (PRRs), leading to activation or prevention of pattern-triggered immunity (PTI), respectively. In addition, specific pathogen effector molecules that are secreted into plant cells are recognized by intracellular nucleotide-binding leucine-rich repeat receptors (NLRs), activating effector-triggered immunity (ETI) [1]. Depending on the pathogen, a mix of PTI, ETI and other immune responses are induced, which are largely mediated by differential accumulation of phytohormones like salicylic acid (SA), jasmonic acid (JA), abscisic acid (ABA), and ethylene (ET) [2]. The different hormones act together in synergistic, antagonistic and additive manners, a phenomenon known as crosstalk [3]. Diverse regulators, interacting with each other in gene regulatory networks, orchestrate the transcriptional reprogramming that results from pathogen recognition. In this review, we refer to all the different immune-related transcriptional reprogramming as the plant immune transcriptome. Mechanistically, the plant immune transcriptome is determined by the coherent control of multiple transcriptional regulatory machineries including transcription factors (TFs), Mediator complex, co-regulators, DNA and RNA modifiers, chromatin remodelers, etc. [4,5]. These molecular components can be directly or indirectly post-transcriptionally modified by kinases/proteases, SUMO/ubiquitin, and second messengers like reactive oxygen species (ROS) and calcium ions (Ca^2+^) [6,7]. The plant immune network is robust enough to resist a pathogen as long as the recognition and immune activation are timely, despite some of the transcriptional machineries being hijacked by the pathogen [1]. Here, we briefly summarize the transcriptional plant targets of pathogen virulence factors, which facilitate our understanding of the plant immune transcriptome. We highlight how multi-scale regulations of transcription and mRNA modulation are accomplished by different proteinaceous components, which determine induction of different sectors in the immune gene regulatory network. Moreover, we highlight the future multi-omics directions to achieve a systems level comprehension of regulation of the plant immune transcriptome.

## The plant immune transcriptome is targeted by pathogens

The timing and efficiency of elicitation of the immune transcriptome is essential for plants to halt pathogens. Of all the cellular components that are involved in transcriptional reprogramming, the role of transcription factors (TFs) in regulating crucial defense responses is best studied. Mutations in TFs including WRKYs, TGAs, NACs, CBP60s/SARD1, ERFs, bZIPs, bHLHs, MYBs, CAMTAs, and TCPs alter plant disease resistance against different pathogens [6,8–13]. Some of these plant TFs are popular targets of pathogens to arrest induced immune responses in plants (reviewed by [14–16]), underpinning their importance for defense. We refer to Table 1 for a summary of effector molecules of different pathogens that target transcriptional regulators, like TFs, transcriptional (co-)activators or repressors, or Mediator subunits. Many of these effectors modulate SA signaling [17,18], JA signaling [19] or SA-JA crosstalk [20–23], leading to enhanced susceptibility to the biotrophic or necrotrophic pathogen at hand.

**Table 1:** Pathogen effectors and their host targets that are involved in transcriptional regulation during plant immunity

Pathogens	Pathogen effectors	Function of host targets	Name of host targets	Host species	References
*Ralstonia solanacearum*	RipAB	Transcription factor	TGAs	*Arabidopsis thaliana*	[124]
*Xanthomonas campestris pv vesicatoria*	XopD	Transcription factor	MYB30	*Arabidopsis thaliana*	[125]
*Xanthomonas campestris pv vesicatoria*	XopS	Transcription factor	WRKY40	*Capsicum annuum*	[23]
*Ralstonia solanacearum*	PopP2	Transcription factor	WRKY	*Arabidopsis thaliana*	[126]
*Pseudomonas syringae*	AvrRps4	Transcription factor	WRKY	*Arabidopsis thaliana*	[127]
*Verticillium dahliae*	VdSCP41	Transcription factor	CBP60g, SARD1	*Arabidopsis thaliana*	[128]
*Pseudomonas syringae*	HopBB1	Transcription factor	TCP14	*Arabidopsis thaliana*	[129]
Phytoplasma	Phyllogen	Transcription factor	MADS-box	*Arabidopsis thaliana*, *Oryza sativa*	[130]
*Hyaloperonospora arabidopsidis*	HaRxL44	Mediator complex	MED19a	*Arabidopsis thaliana*	[21]
*Hyaloperonospora arabidopsidis*	HaRxL21	Transcriptional co-repressor	TPL	*Arabidopsis thaliana*	[131]
*Pseudomonas syringae*	HopZ1, HopX1	Transcriptional repressor	JAZ	*Arabidopsis thaliana*	[20,22]
*Laccaria bicolor*	MiSSP7	Transcriptional repressor	JAZ	*Populus trichocarpa*	[19]
*Pseudomonas syringae*	AvrPtoB	Transcriptional co-activator	NPR1	*Arabidopsis thaliana*	[18]
*Phytophthora capsici*	RxLR48	Transcriptional co-activator	NPR1	*Arabidopsis thaliana*	[17]

This table summarizes some well-studied effectors secreted by different pathogens that hijack diverse transcriptional regulators of the host plant, including transcription factors, and transcriptional (co-) activators and repressors, to facilitate infection.

Another well-studied example of direct interference with plant immune transcription is that of transcription activator-like effectors (TALEs), which are deployed by many plant-pathogenic xanthomonads. TALEs bind to effector binding elements (EBEs) in the promoters of host susceptibility (S) genes that contribute to disease [24,25]. Recently, TALEs Tal2b and Tal2c from *Xanthomonas oryzae* pv. *oryzicola* (*Xoc*) were shown to activate expression of *OsF3H03g*, encoding a 2-oxoglutarate-dependent dioxygenase that negatively regulates SA-related defense and promotes susceptibility against *Xoc* in *Oryza sativa* [26]. Moreover, several TALEs from *Xanthomonas* sp. induce SWEET sugar transporter genes, resulting in an increased availability of sugar for the pathogen, thereby promoting pathogenesis [27,28].

## Regulation of the plant immune transcriptome occurs at multiple scales

**Figure 1:**
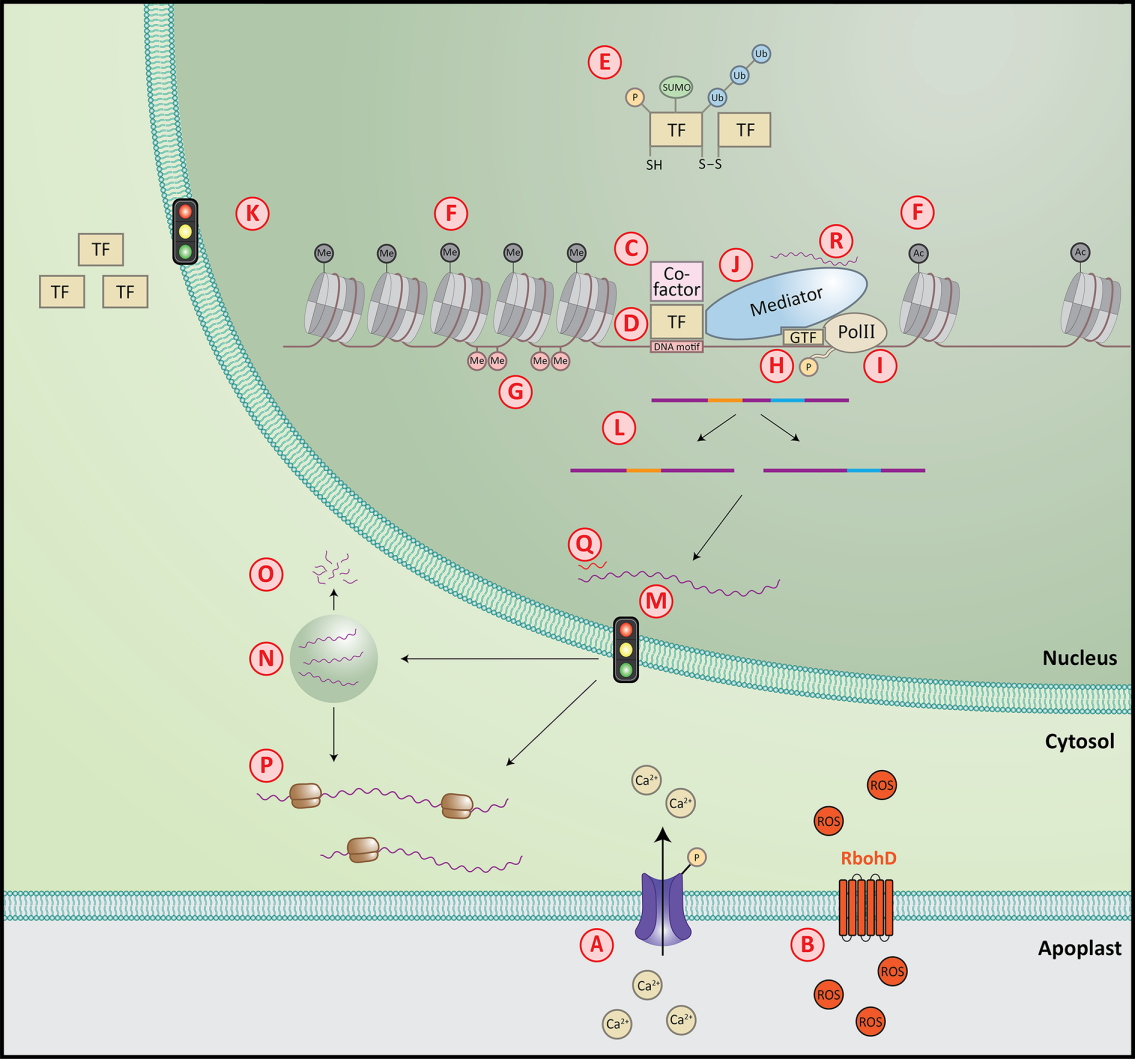
Mechanisms involved in the regulation of immune-related transcription (**A**) Regulation of calcium (Ca^2+^) influx, which may lead to post-translational modifications of TFs (see also Figure 1E); (**B**) generation of ROS by RbohD, which may lead to post-translational modifications of TFs (see also Figure 1E); (**C**) co-factors that may contribute to regulation of transcription; (**D**) TFs regulate transcription by binding to a motif; (**E**) post-translational modifications of TFs, such as phosphorylation (P), sumoylation (SUMO), ubiquitination (Ub) and forming of oligomers through S-S bridges depending on the redox state; (**F**) modifications of histones (methylation [Me] or acetylation [Ac]) to regulate the chromatin state; (**G**) methylation of DNA; (**H**) phosphorylation of the C-terminal domain of RNA-polymerase II (PolII) promotes transcription; (**I**) PolII may initiate transcription at alternative transcription start sites; (**J**) the Mediator complex forms the bridge between specific TFs, general TFs (GTF) and PolII; (**K**) selective import of TFs or other proteins; (**L**) alternative splicing; (**M**) selective retention of mRNAs in the nucleus; (**N**) temporary storage of mRNAs in stress granules or P-bodies; (**O**) degradation of mRNAs from P-bodies; (**P**) release of mRNAs from stress granules or P-bodies into the cytosol, followed by translation; (**Q**) post-transcriptional gene silencing by small RNAs; (**R**) long non-coding RNAs can regulate transcription in different ways, depicted here is modulation of MED19a by ELENA1.

The plant immune transcriptome encompasses both activation and repression of genes with diverse molecular functions, ranging from the control of general metabolic processes to highly specific responses that are directed toward a particular organism [29–37]. The relatively early response to attackers is usually a ‘general stress response’ (GSR) to danger, which is similarly activated after both biotic and abiotic stresses [34,35,38,39], and was demonstrated to be important for defense against *P. syringae* pv. *tomato* DC3000 (*Pto*) [34,35]. Furthermore, for both pathogenic and non-pathogenic bacteria, it was found that the strength of this early response is correlated positively with the number of differentially expressed genes, although it is not clear whether this is based on a causal relationship [35]. The later responses are more specific, depending on the eliciting organism or its derived molecular patterns, and show a high degree of plasticity, which ensures a response tailored to the perceived signal. The transcriptional changes result from multi-scale regulations, including post-translational modifications of TFs, association of TFs with co-regulators and their target DNA sequences, regulation of stability and turnover of TFs, chromatin remodeling, DNA methylation, association of TFs with the Mediator complex, regulation of the RNA polymerases, and post-transcriptional regulation of mRNAs (Figure 1). Below, we highlight some of these mechanisms. We also recommend the recently published focused reviews on TFs functioning in different molecular contexts [40] and epigenetics in plant immunity [41].

### Transcription-related physiological homeostasis

A rapid influx of calcium and a change in redox status are vital parts of plant immunity and they play intertwined roles in PTI and ETI [42]. Calcium influx is induced immediately upon perception of PAMPs and effectors, which has been coupled to classical calcium channels, but also to recently identified noncanonical calcium channels formed by NLR-based resistosomes [43,44] (Figure 1A). Intracellularly, the calcium signal is decoded by calcium-binding proteins like calmodulin (CaM) and Ca^2+^-dependent protein kinases (CDPKs). These can directly activate TFs, such as the defense-regulating CaM-binding TF family CAMTA and CBP60g, or WRKY28, WRKY33 and WRKY48, which are phosphorylated by CPK5 and CPK6 (Figure 1E). This leads to altered defense-related transcription by these TFs, which influences resistance to diverse pathogens [45–48]. Although in general positive effects of calcium signaling on immunity have been reported, this is not always the case. For example, the Ca^2+^-activated CAMTA3 (or AtSR1) TF represses expression of the SA regulator *NPR1* and the SA biosynthesis gene *ICS1* [49]. However, since NPR1 is a negative regulator of HR [50,51], its repressed expression by CAMTA3 positively affects ETI-mediated HR [49]. Moreover, several other CaM-regulated and CaM-like proteins like CBP60a, CML46 and CML47 negatively impact SA-related gene expression and accordingly, mutant lines are enhanced resistant to virulent *P. syringae* [52].

The production and signaling of reactive oxygen species (ROS) is tightly connected to that of calcium, as these molecules can (in)directly regulate each other’s cellular concentrations [42]. Both PTI and ETI trigger a burst of ROS, which is mainly caused by activation of the NADPH oxidase RbohD [53] (Figure 1B). The changed redox state impacts many aspects of a plant’s physiology, including transcription [54]. In plant immunity, NPR1 is the best-known converter of the redox state to transcriptional reprogramming. Under oxidizing conditions, NPR1 resides in the cytosol. According to the classical view it mostly forms oligomers in the cytosol that are held together by disulfide bridges formed between cysteine residues [55], which break under more reduced redox conditions, such as occur during a prolonged defense response, resulting in monomeric NPR1 that relocates to the nucleus [55] (Figure 1E,K). In the nucleus, NPR1 acts as a transcriptional co-activator together with TGA TFs to activate many genes involved in defense [56,57] (Figure 1C,D). The NPR1-TGA1 interaction itself is also affected by the redox status [58]. Interestingly, recent research has challenged the classical literature on the multimerization of NPR1. The oligomeric form of NPR1 in the cytosol that was observed by Mou et al. [55] was found to be likely formed *in vitro* only [59]. However, recently, the cryo-EM structure of NPR1 showed that its predominant functional form is a dimer, which forms oligomers in the quiescent state, but also can interact with two TGA3 dimers to form a TGA3_2_-NPR1_2_-TGA3_2_ complex, and possibly also form complexes with other transcription regulators, to regulate the immune transcriptome [60].

### Post-translational modifications of TFs

Post-translational regulation of TFs can alter their activities (Figure 1E). This is well-studied for the WRKY33 TF that promotes resistance to the necrotrophic pathogen *Botrytis cinerea* by regulating crucial defense-related responses such as camalexin production in *Arabidopsis* [61,62]. The WRKY33 protein is activated by phosphorylation through at least two pathways: one involves the calcium-dependent kinases CPK5 and CPK6 that phosphorylate the Thr-229 residue of WRKY33 [48], and the other involves a MAPK cascade consisting of YDA (a MAPKKK), MKK4 and MKK5 (MAPKKs), and MPK3 and MPK6 (MPKs) that eventually phosphorylate five Ser residues in the N terminus of WRKY33 [48,63–66]. Moreover, SUMOylation of WRKY33 increases its interaction with MPK3 and MPK6, thereby further enhancing WRKY33 phosphorylation via this pathway [67]. The phosphorylation of WRKY33 by the calcium pathway increases its binding to DNA, whereas phosphorylation by the MAPK pathway increases its transactivation activity [48,68]. Genetic studies implied that the same two phosphorylation pathways may also activate MYB51 to regulate indole glucosinolate biosynthesis, but it is not known whether these pathways also play distinct roles in MYB51 functioning [68].

Phosphorylation has also been shown to be important for the transactivation activity and binding specificity to DNA motifs of the ERF TF ORA59, which is required for defense induction in *Arabidopsis* against *B. cinerea* [69]. The hormones JA and ET can induce phosphorylation of ORA59, which binds preferentially to the canonical GCC box or to a newly identified motif named ERELEE4, respectively, depending on the corresponding hormone stimulus [70]. This can explain partly that the ERELEE4 motif is enriched in genes that are induced by ET treatment in an ORA59-dependent manner, while JA treatment is associated with an ORA59-dependent induction of GCC-box containing genes [70].

Ubiquitination also regulates TF activities via protein turnover. For instance, SA induces TF ORA59 ubiquitination and degradation via the 26S proteasome pathway [71,72]. The transcriptional co-regulator NPR1 of SA-induced transcription, and the JAZ repressor proteins and MYC TFs that function in JA-induced transcription, are also regulated by phosphorylation-mediated proteasomal degradation via covalent addition of small ubiquitin proteins [73–75]. Their turnover provides a mechanism to control timing of activation and repression of the plant immune transcriptome. Additionally, SA induces cytoplasmic condensates containing NPR1 and many stress proteins, including specific WRKY TFs and proteins involved in programmed cell death (PCD). NPR1 recruits ubiquitin ligases to these condensates, leading to ubiquitination and subsequent degradation of the proteins and enhanced cell survival during ETI [76].

### Chromatin context

Chromatin context is a major determinant for transcriptional activities in all eukaryotic cells. The accessibility of chromatin can influence when and where TFs, other regulators and RNA polymerases find their targets to activate transcription. The chromatin state can be altered through modification of histone tails and deposition of histone variants (Figure 1F). Recently, Ding et al. (2021) used the method transposase-accessible chromatin followed by sequencing (ATAC-seq) to profile the genome-wide chromatin landscape of *Arabidopsis* after infection with an engineered non-pathogenic *P. fluorescens* strain either expressing the effector AvrRps4 (thus causing both PTI and ETI) or a non-recognized effector mutant (thus causing PTI only), and this was compared with RNA-seq data [77]. Over one-third of all up-regulated genes in both PTI and PTI+ETI also contained more open chromatin compared to the control. Moreover, integration of RNA-seq, ATAC-seq and TF DNA-binding motif information helped to decipher gene regulatory networks mediating PTI, ETI and ‘PTI+ETI’ [77]. In another study, Pardal et al. (2021) used micrococcal nuclease digestion followed by sequencing (MNase-seq) to investigate how treatment with the PAMP flg22 affects genome-wide nucleosome occupancy. They found that flg22 causes genome-wide repositioning of nucleosomes, partly coinciding with the promoters of differentially expressed genes [78]. Repositioning of nucleosomes is mediated by chromatin ATPases [79]. Notably, whereas some chromatin remodeling ATPases like PKR2 and RAD54 promote immunity, others, like EDA16 and SWP73A attenuate it [78,80], indicating a complex relationship between chromatin remodeling and immunity. These studies suggest that chromatin remodeling is an important mechanism by which gene transcription is regulated during immune responses mediated by both cell-surface and intracellular receptors.

It is still unclear whether the accessibility of the regulatory DNA regions precedes transcription or vice versa. It was found that TF WRKY33 enhances accessibility of genes to reinforce gene transcription. The chromatin remodeling complex SWR1 and the MAPK-WRKY33 module promote deposition of H2A.Z [66], a variant of the canonical H2A histone subunit that can activate or repress transcription depending on the context [41], and increased H3K4me3 [66], a histone mark generally associated with active transcription [81]. This happens around WRKY33 target genes, leading to more WRKY33-mediated H2A.Z deposition and H3K4me3 modification [66].

Chromatin remodeling also plays a role during ETI-triggered PCD. During this process chromocenters (regions with heterochromatin) get less dense and different chromatin marks get redistributed, such as the repressive marks H3K9me2 and H3K27me3, leading to altered transcription [82]. Studies with chromatin remodeling mutants suggest that this remodeling mostly attenuates PCD, possibly to prevent it from happening too rapidly or at the wrong time [82].

Chromatin remodeling can also lead to altered transcription via a non-canonical function of the gene-silencing-related component ARGONAUTE1 (AGO1). AGO1 binds to chromatin around specific genes, likely dependent on its association with specific small RNAs and through interaction with several subunits of the SWI/SNF chromatin remodeling complex [83]. There, it promotes PolII occupancy around these genes. Notably, treatment with immune-related compounds such as JA, BTH (an SA analog) and flg22 caused AGO1 to bind to genes that are enriched in GO-terms related to the response to the corresponding ligand, suggesting that AGO1 contributes to these responses. In accordance, a mutation in *AGO1* results in reduced JA-induced gene expression [83].

### DNA methylation

DNA methylation is generally associated with suppression of activity of transposable elements and with transcriptional repression (Figure 1G). DNA methylation in plants can be regulated through RNA-directed DNA methylation (RdDM), which involves small RNAs derived from transcripts resulting from RNA polymerases PolII, PolIV and PolV activity [84]. For examples of regulatory components in RdDM, which shape the immune transcriptome, see also ‘Modulation of RNA polymerase’ and ‘The Mediator complex’.

Demethylation of DNA occurs either passively during replication or actively by different demethylases under specific conditions. The DNA methylome is altered upon pathogen attack, which modulates the immune response [85,86]. The demethylase ROS1 was found to reduce methylation of regulatory regions in or close to flg22-induced defense-related genes, facilitating binding of TFs and subsequent activation of plant immunity [86].

Another example is the demethylase DEMETER (DME). Loss-of-function mutants of this enzyme are lethal, but recent studies using plant lines with a weak allele generated by CRISPR-CAS9 or silencing of *dme* revealed that DME alters methylation of hundreds of genomic regions and is affected in defense against bacterial and fungal pathogens [87]. Moreover, results obtained with the mutant line in which *dme* was silenced in the background of a triple mutant of the DNA methylases *ros1, dml2* and *dlm3* (*rdd*) suggest that DME acts redundantly with other demethylases to regulate expression of defense genes via demethylation [88].

In addition, the demethylation-deficient mutant *rdd* is impaired in resistance against *Pto* induced by the immune-stimulating molecular patterns flg22, elf18 and Pep2 [89]. The flg22 treatment induces hypomethylation of specific regions in the wild-type plant but not in the *rdd* mutant, which is associated with a higher number of differentially methylated promoter regions of defense-related genes and their higher expression level in wild-type compared with mutant. Altogether, the studies discussed here show that DNA demethylation of specific regions is important for a proper immune transcriptome. It has not been explored how the four demethylases change their activity during an immune response, so the spatiotemporal relevance of each enzyme in the regulation of the immune transcriptome remains to be determined.

### Modulation of RNA polymerase

During immune activation PolII is phosphorylated (Figure 1H). For example, flg22 induces phosphorylation of the C-terminal domain (CTD) of PolII [90] by the two cyclin-dependent kinase Cs CDKC;1 and CDKC;2, which in turn are phosphorylated by flg22-triggered MPK3 and MPK6. The phosphatase CTD PHOSPHATASE-LIKE3 (CPL3) can dephosphorylate the CTD and thereby act as a negative regulator of plant immune transcription. Mutants in CDKC and CPL3 were found to be more susceptible to *Pto*, demonstrating the essential role of phosphorylation of the CTD of PolII in regulation of immunity against this pathogen [90]. Another CPL, namely CPL1 of tomato, can reduce defenses against various but not all of the tested pathogens and insects [91]. Additionally, CPL1 negatively regulates defense-related transcription [91]. However, the effect of CPL1 on PolII was not studied, and it even seems likely that other mechanisms are involved, since the *Arabidopsis* homologue of CPL1 has previously been associated with miRNA processing [92] and RdDM [93].

### Alternative transcription initiation

Alternative transcription initiation can expand the regulatory repertoire of the genome, since it may involve alternative promoters that are differentially induced upon different stimuli, or result in different transcripts and proteins (Figure 1I). Recently, more than 15% of the 3374 transcripts that were induced by flg22 treatment after 30 min were found to be derived from alternative transcription events [94]. The alternative transcripts for example lacked upstream open reading frames (uORFs), which may affect translation efficiency of the transcript, or their encoded proteins lacked a predicted domain or signal peptide, which could potentially alter their function. These predictions were validated for a small set of transcripts, but the overall implications of alternative transcription initiation during PTI remain to be elucidated.

### The Mediator complex

TFs recruit PolII through interactions with the multi-subunit Mediator complex [95] (Figure 1J). Recently, substantial molecular evidence has been provided for a role of the Mediator subunit MED25 in hormone crosstalk. The JA-specific TF MYC2 was shown to interact at its same position with the SA regulator NPR1 as well as with MED25 [96]. Consequently, NPR1 reduces the recruitment of MED25 by MYC2 to target promoters of MYC2. This dampens the positive effect of MED25 on MYC2-induced transcription. Interestingly, in the absence of JA, JAZ proteins also repress MYC2-induced transcription in part by preventing the MYC2-MED25 interaction [97]. However, at high JA levels JAZs are degraded [98,99]. NPR1 therefore mechanistically takes over (part of) the function of the degraded JAZ proteins when both JA and SA levels are high.

Although Mediator usually connects TFs to PolII, it can also recruit other RNA polymerases that are relevant for defense. The Mediator subunit MED18 interacts with NUCLEAR RNA POLYMERASE D2 (NRPD2a), a subunit of PolIV and PolV [100]. *MED18* and *NRPD2a* are highly expressed after *B. cinerea* infection, and mutants and overexpressors of these genes corroborate their importance for a part of *B. cinerea*-induced gene expression and for resistance against *B. cinerea* [100,101]. PolIV and PolV are involved in RdDM and other non-coding RNA-mediated gene silencing processes [102], suggesting that impairment of one of these processes may underly the altered gene expression and resistance of the *nrpd2a* mutant and possibly also of the *med18* mutant. However, this still needs to be investigated.

### Selective nuclear transport of transcriptional components

Nuclear im/export of transcriptional components is a selective mechanism to control the plant immune transcriptome (Figure 1K). This was already described for NPR1 in ‘Transcription-related physiological homeostasis’. CONSTITUTIVE EXPRESSER OF PATHOGENESIS-RELATED GENES 5 (CPR5) is a component of the nuclear pore complex that regulates PCD during ETI [103,104]. This protein has three modes of action. Firstly, the conformational change that CPR5 undergoes upon ETI alters the permeability of the nuclear pore and thereby allows influx of several stress-related cargos (such as NPR1 and ABI5) to the nucleus, resulting in massive transcriptional reprogramming [104]. Secondly, CPR5 is a negative regulator of PCD by binding to the cyclin-dependent kinase inhibitors SIAMESE (SIM) and SIAMESE-RELATED1 (SMR1). Upon ETI, CPR5 releases SIM and SMR1, which activate E2F TFs to induce PCD [103]. Finally, CPR5 regulates alternative splicing (AS; Figure 1L) via its RNA-binding activity [105]. Interestingly, the mRNA of the gene-silencing-related *AGO1* and several AS regulators are among its targets, suggesting that apart from its own role in AS, it also indirectly affects gene silencing and AS [105]. In addition, Exportin-4 (XPO4) mediates nuclear export of TOPLESS-RELATED1 (TPR1), counteracting the translocation of TPR1 into the nucleus during ETI in the presence of high SA levels, as was studied in the *cpr5* background [106]. This way, XPO4 prevents the repression of negative immune regulators by TPR1 in the nucleus and probably impedes a runaway immune response during ETI.

### RNA processing, storage, and degradation

Regulation of messenger RNA (mRNA) largely impacts the formation of proteins. For example, AS of NLR genes and JAZ genes generates isoforms with diverse activities or subcellular localizations, by which plants can control immunity activation [107] (Figure 1L). Treatment with flg22 induces MPK4-mediated phosphorylation of splicing factors, leading to AS of genes encoding NLRs, TFs, CDPKs and splicing factors [107]. MED25 recruits the splicing factors PRE-mRNA-PROCESSING PROTEIN 39a (PRP39a) and PRP40a to promote the full splicing of JAZ genes, in order to prevent excessive desensitization of JA signaling mediated by JAZ alternative splice variants [98,99,107,108].

Moreover, RNA can be (temporarily) stored in the nucleus, or in special aggregations that are involved in temporary storage and/or degradation, which decreases the pool of translating RNAs (Figure 1M–P). For example, core hypoxia genes in *Lupinus luteus* and *Arabidopsis* are retained in the nucleus during hypoxia, and released in the cytosol upon reaeration [109]. It is uncertain if such nuclear retention regulation applies during immune activation. Non-translating mRNAs can also be stored in stress granules or P-bodies, which are both quickly disassembled and re-assembled after stress. Stress granules contain mRNAs and translation machinery, and contribute to mRNA storage, whereas P-bodies contain mRNAs and mRNA degrading enzymes, and contribute to mRNA degradation [110,111]. The importance of P-bodies and mRNA decay in PTI has recently been reported [112]. It was shown that the P-body component DECAPPING1 (DCP1), a co-activator of the decapping enzyme DCP2, is phosphorylated by MPK3 and MPK6 within minutes of treatment with several PAMPs. This phosphorylation of DCP1 decreases its binding to DCP2, but increases its binding to XRN4, an exoribonuclease that can degrade decapped mRNA (mRNA decay) [113]. This leads to degradation of a subset of mRNAs that are downregulated during PTI, to prevent their negative impact on the plant immune response [112].

### Regulation by non-coding RNAs

Different non-coding RNAs, like small RNAs and long non-coding RNAs (lncRNAs) regulate different steps in gene expression. For a comprehensive overview on non-coding RNAs in plant immunity we refer to a recent review [114]. Small RNAs can interfere with mRNA stability or regulate transcription or translation through mechanisms such as RdDM (see also ‘DNA methylation’). MicroRNAs (miRNAs) are small RNAs that are involved in post-transcriptional gene silencing (PTGS) and can thus potentially affect the immune transcriptome (Figure 1Q). A recent study explored the role of miRNAs during infection of soybean with the soybean cyst nematode *Heterodera glycines* [115]. They found that differential DNA methylation of miRNA genes influences expression of the miRNAs in resistant and susceptible soybean lines. Overexpression studies show that four miRNAs that are regulated during infection and that are expressed at higher basal levels in a resistant soybean line, cause degradation of their target mRNA and accomplish increased resistance of the susceptible line to the nematode. This study shows that different epigenetic mechanisms (methylation and subsequent miRNA-directed PTGS) interact to finetune the immune response.

A recent study reported that the lncRNA *ELENA1* is induced by treatment with the PAMPs flg22 and elf18 [116]. Overexpression and knockdown studies with *ELENA1* show that it promotes *PR1* and *PR2* expression and resistance to *Pto*. RNA-seq revealed that a subset of elf18-induced defense genes overlaps with ELENA1-induced genes. Upon elf18 treatment, ELENA1 interacts with MED19a *in vivo*, which promotes binding of MED19a to the promoter of *PR1* and possibly other genes [116] (Figure 1R). In a follow-up, it was shown that ELENA1 also mediates the dissociation of the immune suppressor FIBRILLARIN2 from MED19a, providing an additional mechanism by which it can promote target gene transcription [117].

## Multi-omics: challenges and opportunities in studying transcriptional regulation in plant immunity

**Figure 2:**
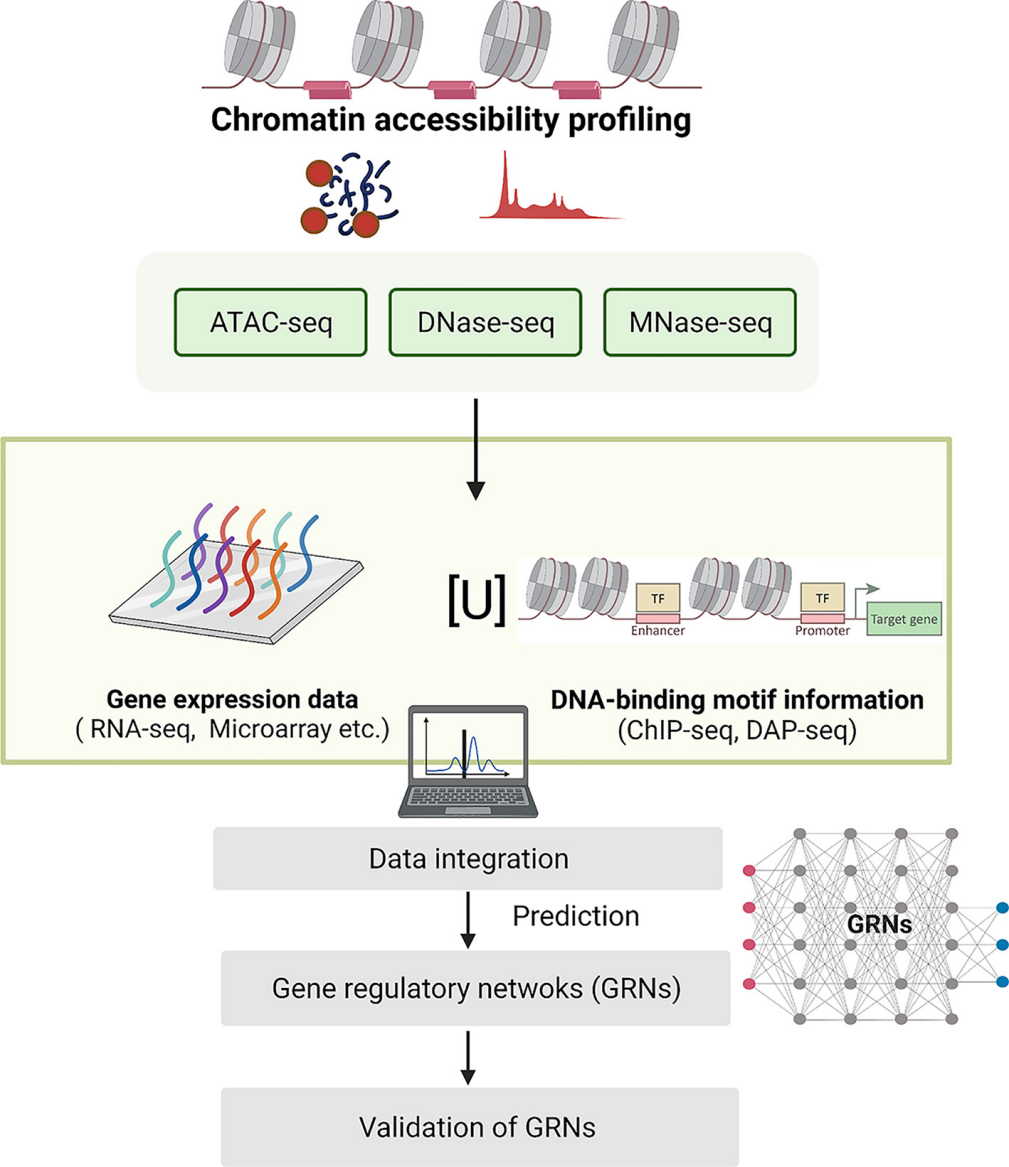
Next-generation toolkit for elucidating immune-responsive GRNs Integration of data on TF DNA-binding, chromatin accessibility, and gene expression can be employed as a powerful tool to elucidate the highly interconnected gene regulatory networks (GRNs) that determine the plant immune transcriptome, even at single cell resolution. For instance, information related to TF-binding sites can be obtained from chromatin-immunoprecipitation followed by sequencing (ChIP-seq), and DNA affinity purification sequencing (DAP-seq). Information about chromatin status can be derived from methods such as Assay for Transposase-Accessible Chromatin using sequencing (ATAC-seq), micrococcal nuclease digestion with deep-sequencing (MNase-seq), or DNase-I hypersensitive sites sequencing (DNase-seq). Different variants of RNA (e.g. mRNA, miRNA, lncRNA) can be measured by RNA-seq. These data can be integrated to reveal GRNs that shape the plant immune transcriptome. The functionality of these GRNs can be tested and validated by mutant analysis under different conditions or in different tissues or cell types.

A balanced regulation of gene expression is required to maintain robustness and efficiency of the triggered immune responses. As outlined in this review, different regulatory components should act in conjunction to control the plant immune transcriptome. How different sectors within the immune network are integrated via these different regulatory players, and under which circumstances, remains largely unknown. A network level understanding could provide leads to these answers. For this, (i) different immune-stimulatory treatments should be compared and (ii) different whole-genome, multi-omics data sets should be combined, (iii) followed by advanced integrated data analysis including the use of mathematical modeling tools (Figure 2). Examples of these omics assays are profiling of chromatin accessibility, RNA variants (mRNA, miRNA, lncRNA, etc), DNA and histone modifications, etc [118–120]. Moreover, the declining costs of nucleotide sequencing, increasing data storage capacities and computer processing power sparks advances in bioinformatic analysis methods and mathematical modeling tools. A systems biology approach will aid in elucidating gene regulatory networks and may provide predictive capability on how and when different regulatory components are involved in orchestrating the plant immune network.

At a finer resolution, namely the cell level, other critical questions need attention. Which of the immune responses are cell-type specific? And does that determine whether the initial infection of a certain cell type propagates further to adjacent cells or is halted? Furthermore, how does cell homeostasis, related to different internal and external conditions, such as plant age, time of day, abiotic stress, and spatiotemporal distance from the infection site, influence the plant immune transcriptome? To answer these questions, single-cell methods instead of bulk analyses using the omics assays and molecular tools mentioned in this review would be extremely meaningful (reviewed by [121]), especially for identifying gene regulatory networks in a heterogenous population from infected to non-infected plant cells. Moreover, analogous profiling of cells of the pathogen will provide insight into the intimate communication between the host and the pathogen [122]. Approaches such as laser microdissection, which has been used widely in clinical biology for studying cell-specific responses [123], can also be used in plant research for molecular profiling of desired cells. With such knowledge collectively, our chances to succeed in intelligently designing crops with a strengthened immune response under diverse conditions will increase.

## Summary

- The plant immune transcriptome is induced upon pathogen perception and required for disease resistance.
- Many pathogens use effectors to tweak the plant immune transcriptome to their own advantage.
- Plants regulate their immune transcriptome at multiple scales, e.g. post-transcriptional regulation of TFs, modulation of DNA accessibility, and modulation of mRNAs during their journey from transcription to translation.
- A combination of multi-omics datasets can provide new insights into immune-related gene regulatory networks.

## Abbreviations

CTD: C-terminal domain
ETI: effector-triggered immunity
NLR: nucleotide-binding leucine-rich repeat receptor
PAMP: pathogen-associated molecular pattern
PCD: programmed cell death
PRR: pattern recognition receptor
PTGS: post-transcriptional gene silencing
PTI: pattern-triggered immunity
uORF: upstream open reading frame
